# Minocycline Alleviates White Matter Injury following Intracerebral Hemorrhage by Regulating CD4^+^ T Cell Differentiation via Notch1 Signaling Pathway

**DOI:** 10.1155/2022/3435267

**Published:** 2022-05-05

**Authors:** Heng Yang, Xinjie Gao, WeiPing Xiao, Jiabin Su, Yanjiang Li, Wei Ni, Yuxiang Gu

**Affiliations:** ^1^Division of Cerebrovascular Surgery and Interventional Neuroradiology, Department of Neurosurgery, Huashan Hospital, Fudan University, China; ^2^Neurosurgical Institute, Fudan University, China; ^3^Shanghai Clinical Medical Center of Neurosurgery, China; ^4^National Center for Neurological Disorders, China

## Abstract

Neuroinflammation is a major reason for white matter injury (WMI) after intracerebral hemorrhage (ICH). Apart from microglia/macrophage activation, T cells also play an important role in regulating immune responses after ICH. In a previous study, we have revealed the role of minocycline in modulating microglia/macrophage activation after ICH. However, the exact mechanisms of minocycline in regulating T cells differentiation after ICH are still not well understood. Hence, this study explored the relationship between minocycline and CD4^+^ T cell differentiation after ICH. Piglet ICH model was used to investigate naive CD4^+^ T cell differentiation and T cells signal gene activation after ICH with immunofluorescence and whole transcriptome sequencing. Naive CD4^+^ T cells and primary oligodendrocyte coculture model were established to explore the effect and mechanism of minocycline in modulating CD4^+^ T cell differentiation after ICH. Flow cytometry was used to indicate CD4^+^ T cell differentiation after ICH. The mechanism of minocycline in modulating CD4^+^ T cell differentiation was demonstrated with immunofluorescence and western blot. Double immunostaining of representative CD4^+^ T cell marker CD3 and different subtype CD4^+^ T cell assisted proteins (IL17, IL4, Foxp3, and IFN*γ*) demonstrated naive CD4^+^ T cell differentiation in piglet after ICH. Whole transcriptome sequencing for perihematomal white matter sorted from piglet brains indicated T cell signal gene activation after ICH. The results of luxol fast blue staining, immunofluorescent staining, and electron microscopy showed that minocycline alleviated white matter injury after ICH in piglets. For our *in vitro* model, minocycline reduced oligodendrocyte injury and neuroinflammation by regulating CD4^+^ T cell differentiation after ICH. Moreover, minocycline increased the expression of NOTCH1, ACT1, RBP-J, and NICD1 in cultured CD4^+^ T cell when stimulated with hemoglobin. Hence, minocycline treatment could modulate naive CD4^+^ T cell differentiation and attenuate white matter injury via regulating Notch1 signaling pathway after ICH.

## 1. Introduction

Intracerebral hemorrhage (ICH) is a subtype of stroke that is associated with high mortality and disability. It can cause gray matter injury and damage to the white matter region. White matter is located at the subcortical area and composed mostly of bundled axons and glia cells, including myelin-producing oligodendrocytes, astrocytes, and microglia. WMI is related to sensorimotor deficit and cognitive impairment after stroke [1]. Our previous research has revealed that severe white matter edema and disruption in experimental ICH model resulted in serious neurological dysfunction [2, 3]. Therefore, ICH-induced white matter injury (WMI) is one of the major reasons for poor prognosis in ICH patients. Although hematoma evacuation and pharmacological interventions are used in the treatment of ICH, there is still a lack of effective therapy for patients with ICH due to the complex pathophysiological mechanisms of WMI following ICH.

Multiple mechanisms are involved with WMI after stroke, including demyelination, axon degeneration, and neuroinflammation [4–6]. ICH is known to induce a complex array of inflammatory responses, including blood-brain barrier disruption, and infiltration of immune cells such as leukocytes, T cells, macrophages, and natural killer cells which causes both gray matter and white matter injury. In addition, the resident microglia activation also acts as an important factor in post-ICH inflammatory cascades. In a previous study, we have demonstrated the role of macrophage/microglia activation in white matter injury and repair following ICH [7, 8]. It has been established that T helper (Th) cells and T regulatory cells (Treg) also play an essential role in regulating immune response and correlate with brain damage and repair after ICH [9]. However, the modulatory effect of T cells in white matter injury and repair after ICH is not well understood. Recently, the different roles of T cells in demyelination and remyelination have been demonstrated. Th cells play an important role in regulating immune response by secretion of different cytokines, including interleukin- (IL-) 17, IL-21, and IL-22, and inducing inflammation after ICH. Studies demonstrated the increase of Th 17 cells could exaggerate inflammation after ICH [10]. However, depleting the IL-17 could modulate the inflammatory environment to enhance myelin regeneration and prevent axonal injury. Treg cells possess the ability to protect against inflammatory injury after ICH by inhibiting microglia activation [11] and promoting oligodendrocyte differentiation and remyelination in the central nervous system [12]. Therefore, regulating the functional differentiation of CD4^+^ T cells and the balance of Th17/Treg cells ratio may provide a novel and direct insight for white matter repair following ICH.

The Notch signaling pathway is an important regulator for cellular differentiation and immune response. Studies have demonstrated that the Notch signaling pathway could regulate the differentiation of naive CD4^+^ T cells [13]. Inhibiting the Notch signaling can reverse the Th17/Treg cellular imbalance, reduce the inflammatory response, and enhance oligodendrocyte progenitor cell proliferation and differentiation [14, 15]. Our previous studies revealed that minocycline can reduce WMI after ICH via anti-inflammatory mechanism. Although the studies have validated the role of minocycline in regulation of the Notch signaling, there are more to uncover on the mechanisms of minocycline in regulating CD4^+^ T cell differentiation following ICH. For this present study, we investigated a novel mechanism of Notch signaling-mediated naive CD4^+^ T cell differentiation and demonstrated the effects of minocycline in white matter repair after ICH via regulation of CD4^+^ T cell differentiation.

## 2. Materials and Methods

### 2.1. Animal Preparation and Intracerebral Hemorrhage Model

Piglets (15-25 kg, 4-5 months) were obtained from the Experimental Animal Center of Shanghai Jiaotong University. The protocol involving animals was approved by the Animal Ethics Committee of Fudan University. A total of 9 male piglets were used in this study. The ICH models were performed as previously described [2, 16]. Before the operation, the animals were acclimatized to the surroundings for at least one week. Animals were sedated with ketamine (15-20 mg/kg, I.M.) and diazepam (5-10 mg/kg, I.M.) for induction of anesthesia and endotracheal intubation. Then, 5% pentobarbital sodium was used to maintain anesthesia during the surgical procedures. Body temperature was maintained at 37.5 ± 0.5°C by the heating pad. The right femoral artery was catheterized with a polyethylene catheter (PE-160) to obtain blood for injection and to monitor arterial blood pressure and arterial blood gases. A cranial burr hole (1.5 mm) was then drilled 11 mm to the right of the sagittal suture and 11 mm anterior to the coronal suture. An 18.5 mm long 18-gauge sterile plastic catheter was placed into the center of the right frontal cerebral white matter at the level of the caudate nucleus. First, 1 mL of autologous arterial blood was infused for 10 min with an infusion pump. Then, another 1.5 mL of blood was also injected for 10 min after a 5 min break [2]. Sham piglets underwent the same procedure without blood infusion. Piglets were divided into three groups (*n* = 3/group). Piglets in the experimental group were treated with minocycline (4 mg/kg, intramuscular injection, at 2 hours after ICH and then 2 mg/kg, every 12 hours for 3 days). Animals in the control and sham groups were treated with vehicle.

### 2.2. Transcriptional Analysis

Piglet brain tissue in sham and ICH group was used for RNA-seq. RNA isolation and quantification were performed according to the protocols in previous reports [17], and sequencing of the total RNA profile was performed using a HiSeq 4000 (Illumina, USA). Transcriptional analysis was performed as described previously [18]. In brief, heatmaps were used to demonstrate inflammatory response-related genes expression and transcriptional profiles of key cytokines in CD4^+^ T cell differentiation after ICH. Pathway-pathway network analysis was then performed to identify potential interaction networks using Cytoscape 3.4.0 (Agilent and IBS).

### 2.3. Immunofluorescence Staining

Paraffin-embedded brains were sliced into 10 *μ*m thick sections. The sections were deparaffinized in xylene and rehydrated in a graded series of alcohol dilutions. Antigen retrieval was performed by the microwave method using citrate buffer (10 mM, pH 6.0). All sections were then treated with 0.3% hydrogen peroxide to neutralize endogenous peroxidases. Sections were then blocked with 3% bovine serum albumin (BSA) in 0.1% Triton X-100 (*v*/*v*) for half an hour at room temperature and washed three times in 0.1 mol/L phosphate-buffered saline (PBS, pH 7.4). The sections were incubated with specific primary antibodies at 4°C overnight. Following overnight incubation, sections were rinsed with PBS and incubated at room temperature for 1 h with secondary antibodies. The primary antibodies were monoclonal rat antimyelin basic protein (Abcam, 1: 500), monoclonal rabbit anti-CD3 (Abcam, 1: 200), monoclonal rabbit anti-CD4 (Abcam, 1: 1000), polyclonal rabbit anti-IL17A (Abcam, 1: 100), monoclonal mouse anti-Notch1 (SANTA CRUZ, 1: 100), monoclonal mouse anti-IL4 (Abcam,1: 100), monoclonal rabbit anti-Foxp3 (CST, 1: 100), and polyclonal rabbit anti-IFN*γ* (Proteintech, 1: 100). Secondary antibodies were Alexa Fluor 488 donkey anti-mouse IgG (Jackson, 1: 1000), Alexa Fluor 488 donkey anti-rabbit IgG (Jackson, 1: 1000), and Alexa Fluor 594 donkey anti-rabbit IgG (Jackson, 1: 1000).

### 2.4. Luxol Fast Blue Staining

Luxol fast blue staining was performed using Luxol fast blue-cresyl echt violet stain kit (American Mastertech). Brain sections were dehydrated with alcohols and then incubated in the Luxol fast blue stain solution at 60°C overnight, followed by washing with distilled water. Sections were then quickly dipped in 0.05% lithium carbonate and 70% regent alcohol for gray and white matter differentiation. Then, the slices were incubated in cresyl echt violet stain for 10 minutes, dipped in 70% regent alcohol for 5–10 times, dehydrated through three changes of absolute alcohol, and finally mounted with Permount (Fisher Scientific). The white matter bundles were examined by light microscopy.

### 2.5. Western Blotting

Western blot analysis was performed as previously described [3]. White matter tissue adjacent to the hematoma was sampled. The primary and secondary antibodies were polyclonal rabbit anti-IL17A (Abcam, 1: 1000), polyclonal rabbit anti-IL21 (1: 500, Abcam), polyclonal rabbit anti-IL22RA2/IL-22BP (Abcam, 1: 1000), monoclonal rabbit anti-TGF-*β*1 (Abcam, 1: 1000), monoclonal rabbit anti-TNF*α* (Abcam, 1: 1000), polyclonal rabbit anti-IFN*γ* (Proteintech, 1: 500), monoclonal rabbit anti-Notch1 (Abcam, 1: 1000), polyclonal rabbit anti-ACT-1 (Proteintech, 1: 1000), monoclonal rabbit anti-RBP-J (Abcam, 1: 1000), and monoclonal rabbit anti-NICD1 (CST, 1: 1000).

### 2.6. Real-Time PCR

RT-PCR was performed as described previously [19]. Briefly, total RNA was isolated with TRIzol reagent (Thermo Fisher Scientific), then, RNA was reverse-transcribed into cDNA using the Superscript First-Strand Synthesis System (Invitrogen, Grand Island, NY, USA). RT-PCR was performed using the Opticon2 Real-Time PCR Detection System (Bio-Rad) and SYBR gene PCR Master Mix (Invitrogen). Cycle time values were measured as a function of *GAPDH* mRNA levels in the same tissue. The sequences of the primer pairs for the inflammatory cytokines are listed in Table 1.

### 2.7. Electron Microscopy

Electron microscopy was performed as described previously [20] to access myelin and axons damages in the peri-hemorrhage area. Briefly, piglets were perfused with saline, followed by ice-cold 4% paraformaldehyde and 0.1% glutaraldehyde in 0.1 mol/L PBS (pH 7.4). White matter tissue in the peri-hematoma area was microdissected into 1 mm blocks and fixed in 2% glutaraldehyde overnight. Then, the tissues were washed in 0.1 mol/L sodium cacodylate buffer (pH 7.4), postfixed in buffered osmium tetroxide for 1–2 h. Following serial dehydration in acetone, the tissue was embedded in epoxy resin. Sections of 60–90 nm thicknesses were placed onto 200 mesh grids, stained with uranyl acetate and lead citrate, and then were examined with a JEOL JEM-1230 transmission electron microscope. We calculated the G-ratio (axonal diameter with myelin sheath/axonal diameter without myelin sheath) to assess white matter injury after ICH. Two consecutive sections from each animal at peri-hematoma area were analyzed. Two images were acquired in randomly selected areas within the peri-hematoma from each section and analyzed with ImageJ by an investigator blinded to experimental groups.

### 2.8. Naive CD4^+^ T Cells and Primary Oligodendrocyte Cultures

Naive CD4^+^ T cells were collected from healthy rats, and primary oligodendrocytes were from mixed cultures harvested from one- to two-day-old postnatal rats, as described previously [21]. Oligodendrocytes were cultured in medium containing 15 nM triiodothyronine and 1 ng/mL ciliary neurotrophic factor. CD4^+^ T cells were treated with PBS, minocycline (40 *μ*M), hemoglobin (20 *μ*M) + PBS, and hemoglobin (20 *μ*M) + minocycline (40 *μ*M), respectively. Hemoglobin was added to CD4^+^ T cells for 6 h, and then CD4^+^ T cell-conditioned media was transferred from CD4^+^ T cell cultures to oligodendrocyte cultures via a transwell system. The following assays were performed 24 h later: cell counting kit-8 (CCK-8) assay for viability, lactate dehydrogenase (LDH) activity assay for loss of membrane integrity, and immunocytochemical staining for Myelin basic protein (MBP).

### 2.9. Frequencies of Th1, Th2, Th17, and Treg Cells after Minocycline Intervention in In Vitro ICH Model

To explore the effect of minocycline (mino) on naive CD4^+^ T cell differentiation after ICH, we further measured the numbers of Th1, Th2, Th17, and Treg cells after minocycline intervention. Briefly, CD4^+^ T lymphocytes from healthy rats were divided into T cell + OPCs group, T cell + OPCs+ Mino group, T cell+ OPCs+ hemoglobin group, and T cell + OPCs+ hemoglobin + Mino group. T lymphocytes from each tissue were seeded in 24-well flat plates (1.0 × 10^6^ cells/ml.cm^2^) and were polarized under Th17 cell-polarizing cocktail consisting of anti-rat CD3 (5 *μ*g/mL), anti-rat CD28 (2 *μ*g/mL), rIL-1*β* (20 ng/mL), rIL-6 (60 ng/mL), rIL-23 (30 ng/mL), and TGF-*β* (2 ng/mL). All cells were cultured at 37°C for 72 h in a humidified incubator with 5% CO_2_. At the indicated time, the numbers of Th1, Th2, Th17, and Treg cells were measured by flow cytometry (BD FACSVerse™, USA). Meanwhile, an isotype control assay was also performed to eliminate the background from cell staining.

### 2.10. Statistical Analysis

All data in this study are presented as the mean ± standard error of the mean (SEM) and have been analyzed using the SPSS 22.0 software. Student's *t*-test was used to analyze differences between two groups, whereas differences between multiple groups were analyzed with one-way analysis of variance (ANOVA). Two-way ANOVA was used to evaluate differences in the behavior tests between groups and between time points. Differences were considered statistically significant when *P* < 0.05.

## 3. Results

### 3.1. Minocycline Alleviated White Matter Injury after ICH in Piglets

**Figure 1:**
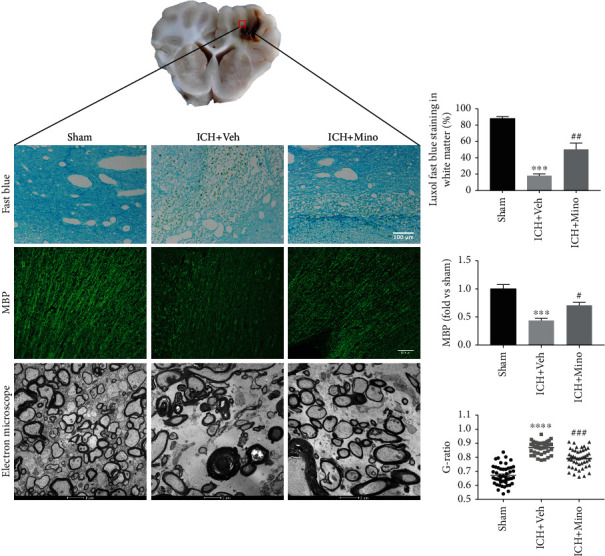
Minocycline alleviated white matter injury after ICH in piglets. Luxol fast blue, immunostaining, and electron microscopy images demonstrated that minocycline reduced white matter damage around the hematoma after ICH.

The effect of minocycline in WM protection after ICH was investigated using Luxol fast blue staining, immunofluorescent staining, and electron microscopy. The techniques demonstrated white matter injury in the sham, ICH + Mino, and ICH + Veh groups, respectively (Figure 1). The Luxol fast blue staining showed less white matter injury in the ICH + Mino group compared with the ICH + Veh group whereas the immunofluorescent staining of myelin basic protein (MBP) indicated that minocycline alleviated the MBP reduction after ICH. In addition, electron microscopy showed demyelination of WM regions in ICH piglets, but minocycline treatment preserved the myelin sheaths via the prevention of demyelination at 14 days post-ICH.

### 3.2. ICH Induced CD4^+^ T Cell Differentiation and T Cell Signal Gene Activation in Piglet ICH Model

**Figure 2:**
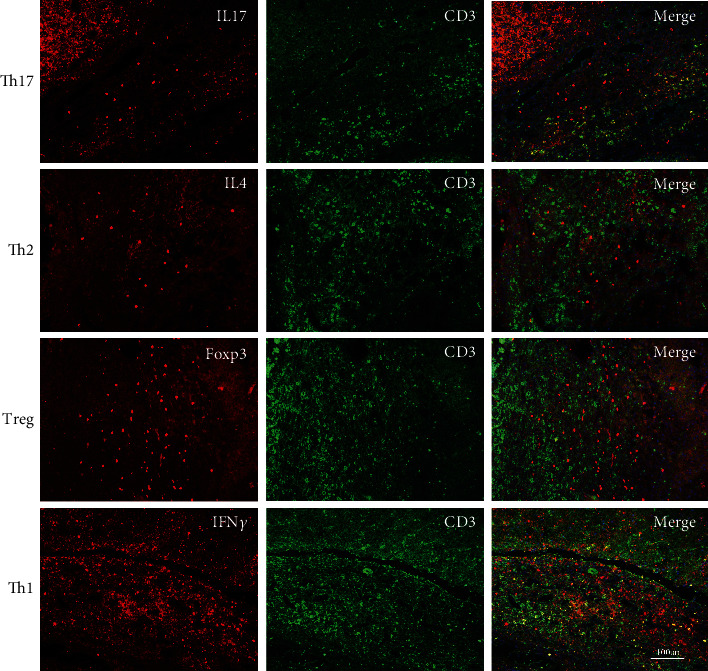
Differentiation of CD4^+^ T cell after ICH in piglets. Double immunofluorescence staining of CD3 with IL17, IL4, Foxp3, and IFN*γ*, respectively, demonstrated that CD4^+^ T cells differentiated to Th1 and Th17 cells after ICH.

Double immunostaining of representative CD4^+^ T cell marker CD3 and different subtype CD4^+^ T cell assisted proteins (IL-17, IL4, Foxp3, and IFN*γ*) were performed to specifically evaluate the CD4^+^ T cell differentiation in piglet after ICH (Figure 2). We observed that naive CD4^+^ T cells were activated and differentiated into different phenotypes, including T-helper 1, T-helper 2, T-helper 17, and regulatory T cells. The activation and differentiation were especially prominent in Th17 and Th1 cells.

**Figure 3:**
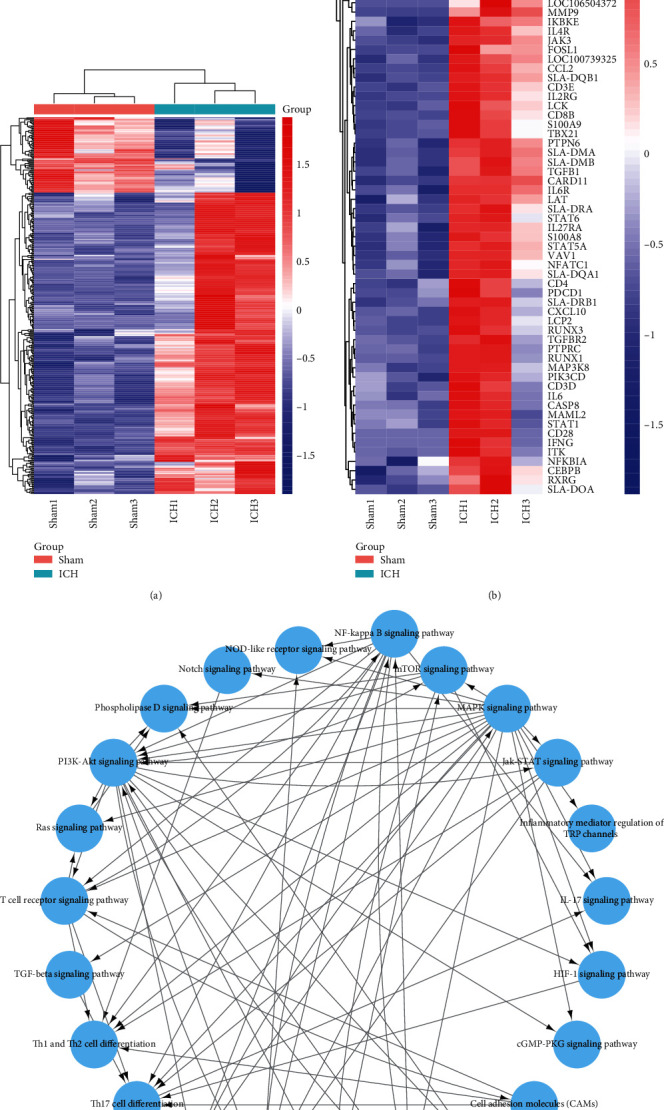
T cells signal gene activation in piglet ICH model. (a) Heatmap of inflammation-related genes expression in sham and ICH piglets. (b) Transcriptional profiles of key cytokines in CD4^+^ T cell differentiation (*n* = 3). (c) Interaction network diagram (pathway-pathway network) indicated the signal pathway of T cell differentiation after ICH.

To detect the profiles of neuroinflammatory gene expression in white matter after ICH, we performed whole transcriptome sequencing for perihematomal white matter sorted from piglet brains over the course of three days. When comparing with the sham group, there were more than 2000 differentially expressed genes in the ICH group at day three postoperation. The heat map showed an increased expression of inflammation-related genes at day three after ICH (Figure 3(a)). The T cell activation and differentiation-related signals were selected and analyzed. The heat map shown in Figure 3(b) demonstrated an increased expression of T cell activation and differentiation-related genes after ICH. In addition, to investigate the potential mechanisms of ICH-induced CD4^+^ T cell differentiation, the pathway-pathway network of T cell activation and differentiation was analyzed, indicating activation of several signal pathways related to T cell differentiation, including the Notch signaling pathway (Figure 3(c)).

### 3.3. Minocycline Reduced Oligodendrocyte Injury and Neuroinflammation by Regulating CD4^+^ T Cell Differentiation after ICH In Vitro

**Figure 4:**
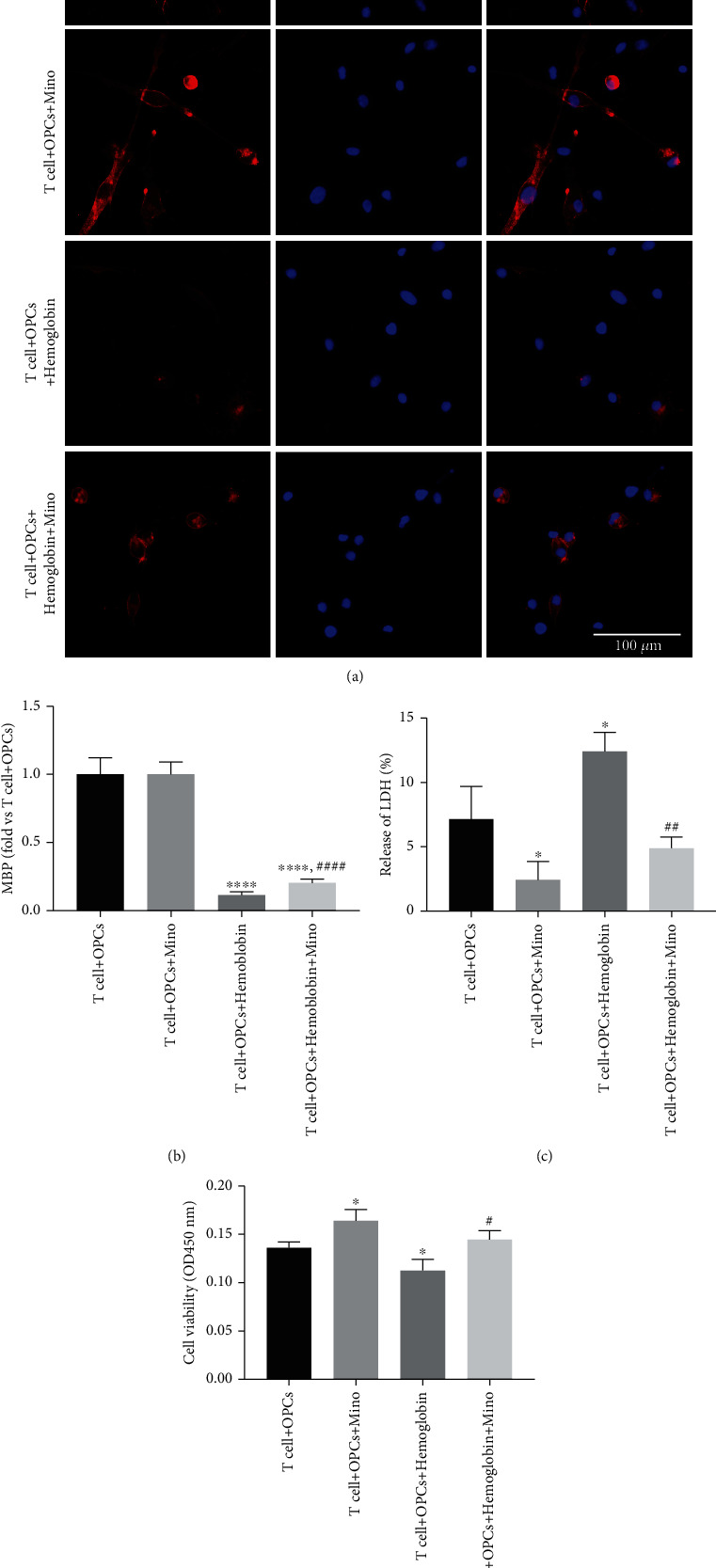
Minocycline reduced oligodendrocyte injury after ICH in vitro. (a) Representative images of immunostaining assays for white matter marker MBP in the T cell + OPCs, T cell + OPCs + Mino, T cell + OPCs + hemoglobin, and T cell + OPCs + hemoglobin + Mino groups. (b, c) Quantifications of oligodendrocyte survival and cell death with LDH release and CCK8 in the transwell system.  ^∗^*P* < 0.05 vs. the T cell + OPCs group; ^#^*P* < 0.05, ^##^*P* < 0.01 vs. the T cell + OPCs + hemoglobin group.

We further investigated the relationship between CD4^+^ T cell differentiation and white matter injury. Naive CD4^+^ T cells were stimulated with hemoglobin and cocultured with oligodendrocytes in a transwell system to evaluate the minocycline-mediated protection of oligodendrocytes. From the medium of cultured CD4^+^ T cells that were stimulated by hemoglobin, a decreased immunostaining intensity of MBP was observed, indicating damage to the cultured oligodendrocytes. However, minocycline inhibited the decrease of MBP immunostaining intensity (Figure 4(a)). In addition, minocycline could also protect oligodendrocyte viability (Figures 4(b) and 4(c)). This was reflected by the results from the assays such as LDH release and CCK8. Hence, it was suggested that minocycline could protect oligodendrocyte injury after ICH.

**Figure 5:**
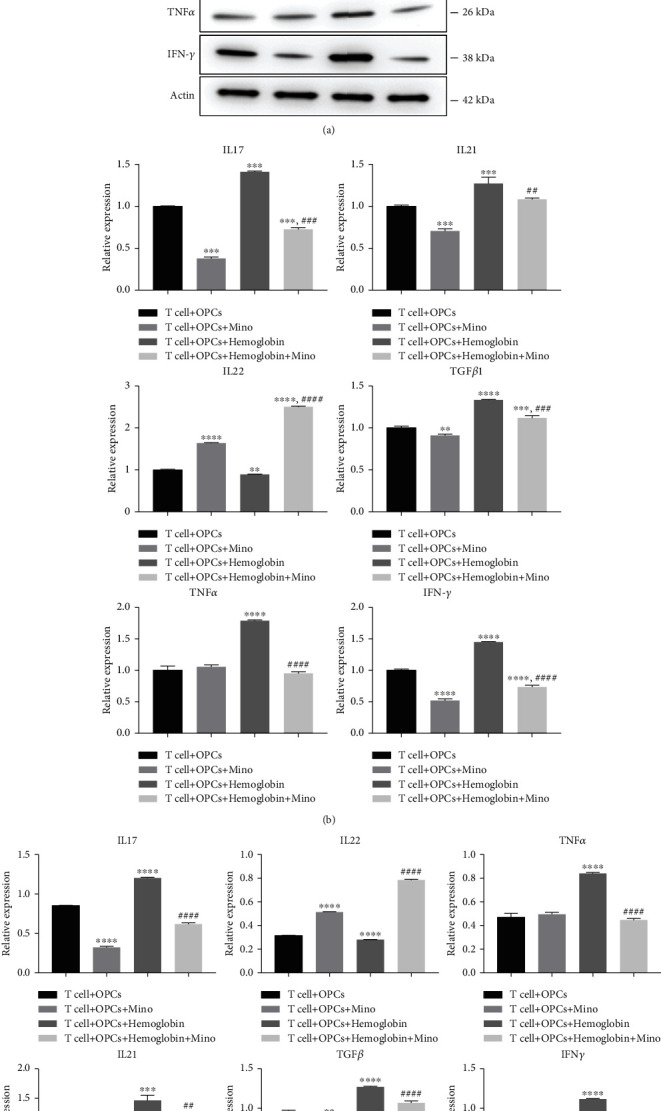
Minocycline reduced neuroinflammation after ICH *in vitro*. (a) Minocycline reduced the protein levels of IL17, IL21, IL22, TGF*β*, TNF*α*, and IFN*γ* after hemoglobin stimulation. (b) Changes of IL17, IL21, IL22, TGF*β*, TNF*α*, and IFN*γ* mRNA levels in the T cell + OPCs group, T cell + OPCs + Mino group, T cell + OPCs + hemoglobin group, and T cell + OPCs + hemoglobin + Mino group.  ^∗^ ^∗^*P* < 0.01,  ^∗^ ^∗^ ^∗^*P* < 0.001,  ^∗^ ^∗^ ^∗^ ^∗^*P* < 0.0001 vs. the T cell + OPCs group; ^##^*P* < 0.01, ^####^*P* < 0.0001 vs. the T cell + OPCs + hemoglobin group.

Thereafter, the expression of proinflammatory cytokines in CD4^+^ T cells was assessed to indicate the anti-inflammatory effect of minocycline in *in vitro* ICH model. The relative protein and mRNA levels of IL17, IL21, TGF*β*, TNF*α*, and IFN*γ* were increased due to hemoglobin stimulation. However, minocycline treatment inhibited the increase of proinflammatory cytokines after ICH (Figure 5). These results suggested that activated CD4^+^ T cells may induce white matter injury by secreting proinflammatory cytokines after ICH and that minocycline could inhibit neuroinflammation and reduce white matter injury after ICH.

**Figure 6:**
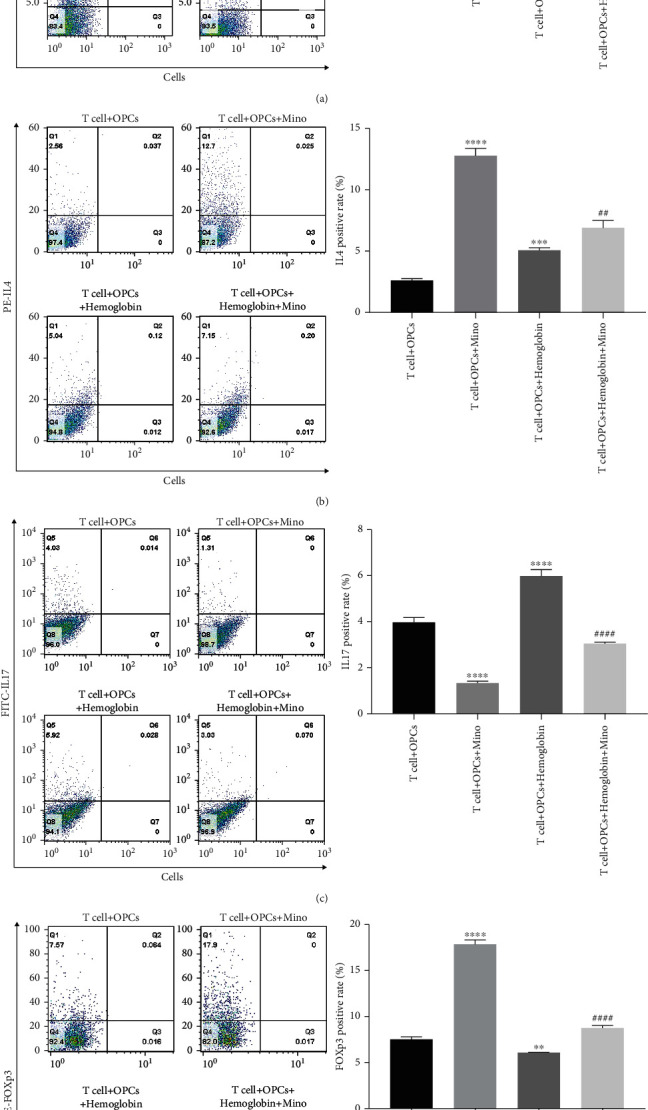
Minocycline regulated CD4+ T cell differentiation after ICH. Frequencies of Th1 cells (a), Th2 cells (b), Th17 cells (c), and Treg cells (d) in the T cell + OPCs group, T cell + OPCs + Mino group, T cell+ OPCs + hemoglobin group, and T cell + OPCs + hemoglobin + Mino group were detected by flow cytometry.  ^∗^ ^∗^*P* < 0.01,  ^∗^ ^∗^ ^∗^*P* < 0.001,  ^∗^ ^∗^ ^∗^ ^∗^*P* < 0.0001 vs. the T cell + OPCs group; ^##^*P* < 0.01, ^####^*P* < 0.0001 vs. the T cell + OPCs + hemoglobin group.

We have also investigated the relationship between minocycline and CD4^+^ T cell differentiation after ICH *in vitro*. We applied a gating strategy to sort CD4^+^ T cells by flow cytometry. After culturing CD4^+^ T cells for 72 h after hemoglobin stimulation, it was noted that the frequency of CD4^+^ T cell subsets and Th1 (or Th17) cells in T cells + OPCs + hemoglobin group was significantly higher than that in the T cells + OPCs + hemoglobin + Mino group (Figures 6(a) and 6(c)). Nevertheless, the frequency of CD4^+^ T cell subsets and Th2 (or Treg) cells in T cells+ OPCs+ hemoglobin group was significantly lower than that in the T cells+ OPCs+ hemoglobin + Mino group (Figures 6(b) and 6(d)). These results indicated that minocycline could regulate CD4^+^ T cell differentiation after ICH and improve CD4^+^ T cell differentiation to Th2 and Treg cells, while inhibiting the differentiation of CD4^+^ T cell into Th1 and Th17 cells.

### 3.4. Minocycline Activated the Notch1 Signaling in CD4^+^ T Cells after ICH

**Figure 7:**
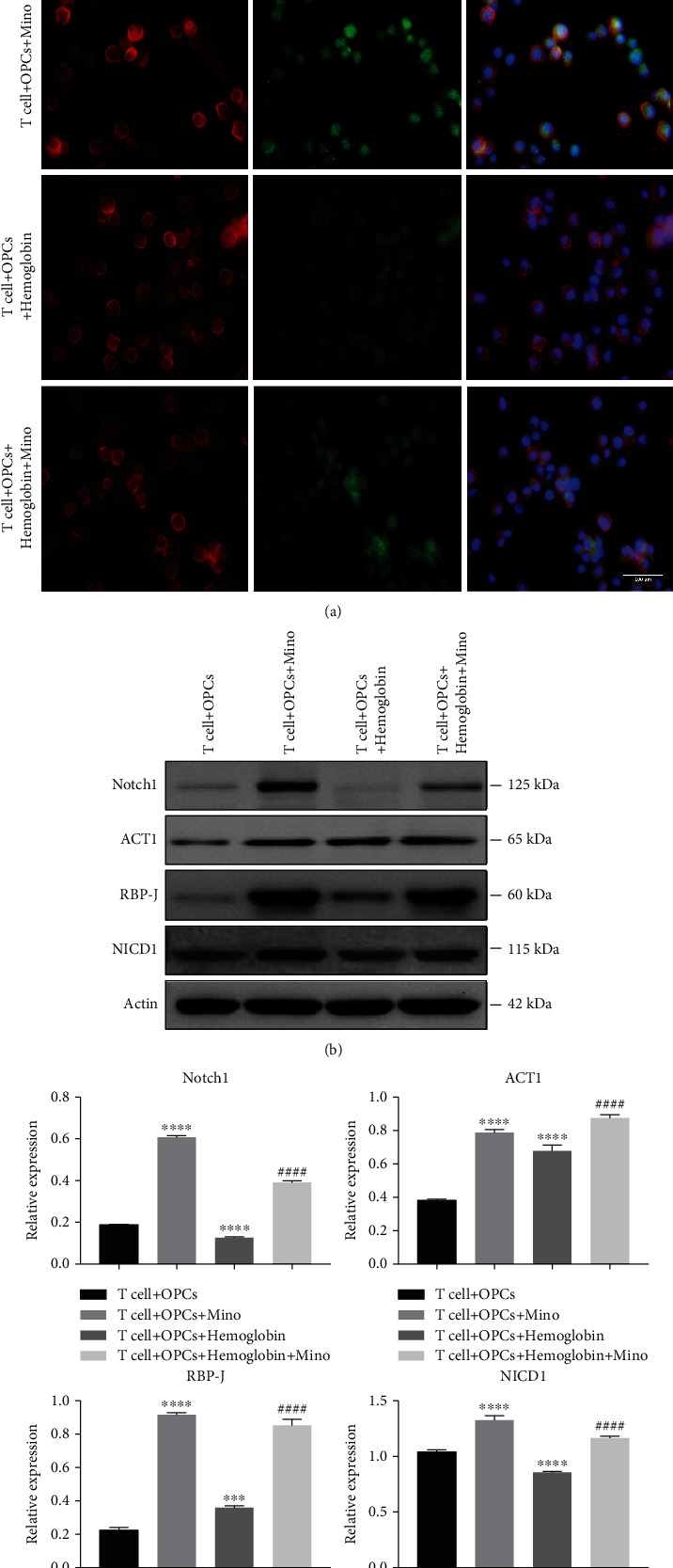
Minocycline activated the Notch1 signaling in CD4^+^ T cells after ICH. (a) Representative immunostaining images of CD4 and Notch1 in different groups. (b) CD4^+^ T cells were cultured *in vitro* with PBS, minocycline, hemoglobin + PBS, and hemoglobin + Mino to check the protein levels of Notch1 signal. (c) Quantification of protein levels of Notch1 signal in different groups.  ^∗^ ^∗^ ^∗^*P* < 0.001,  ^∗^ ^∗^ ^∗^ ^∗^*P* < 0.0001 vs. the T cell + OPCs group; ^####^*P* < 0.0001 vs. the T cell+ OPCs+ hemoglobin group.

The potential mechanism of minocycline in modulating CD4^+^ T cell differentiation and white matter protection after ICH was evaluated by performing a double immunostaining of CD4 and Notch1 in the T cell + OPCs, T cell + OPCs + Mino, T cell + OPCs + hemoglobin and T cell + OPCs + hemoglobin + Mino groups, respectively. Results from the immunostaining demonstrated that minocycline elevated the expression of Notch1 in CD4^+^ T cells after ICH (Figure 7(a)). Moreover, the protein levels of Notch1, ACT1, RBP-J, and NICD1 in the T cell +OPCs+ hemoglobin group were all significantly lower than those in the T cell +OPCs+ hemoglobin+ Mino group (Figures 7(b) and 7(c)). Therefore, minocycline could improve the Notch1 signal activation after ICH.

## 4. Discussion

In this study, we investigated whether minocycline treatment could alleviate white matter injury after ICH via modulation of CD4^+^ T cell differentiation. The key findings of our study are [1] CD4^+^ T cell differentiation has been observed in white matter injury after ICH; [2] minocycline treatment could reduce neuroinflammation and white matter injury by improving CD4^+^ T cell differentiation to Th2, Treg cells and inhibiting CD4^+^ T cell differentiation to Th1, Th17 cells after ICH; and [3] minocycline regulates CD4^+^ T cell differentiation via Notch1 signaling after ICH.

White matter injury can result in poor prognosis of patients with ICH. Several studies have suggested that neuroinflammation plays an important role in secondary injury of white matter after ICH [6]. Neuroglia cell activation is known to be one of the underlying causes of neuroinflammation following ICH [22, 23]. We have previously confirmed that neuroinflammation following macrophage/microglia activation is considered a double-edged sword that can exert either neuroprotective or neurotoxic effects after ICH [22]. Apart from glia cell activation, T lymphocytes could also contribute to inflammation, thereby exacerbating brain injury after ICH [10, 24]. In ICH patients, CD4^+^ and CD8^+^ T cells migrate into the brain and promote cerebral inflammation and brain injury. However, certain types of T cells could also exert neuroprotective activity after ICH. It has been established that CD4^+^ T cells are a type of helper T cells, which can modulate immune response. When they are activated, CD4^+^ T cells differentiate into four types of T helper (Th) cells: Th1, Th2, Treg, and Th17 cells. On one hand, Th1 and Th17 cells could activate macrophage/microglia activity and contribute to inflammation [25]; whereas, Th2 and Treg cells are critical for anti-inflammatory activity and repair. However, the relationship between T lymphocyte differentiation and white injury after ICH is still unknown. In this study, both the *in vivo* and *in vitro* ICH models demonstrated CD4^+^ T cell differentiation is associated with white matter injury after ICH. Therefore, the modulation of CD4^+^ T cell differentiation may be a potential therapeutic target for the treatment of white matter injury after ICH.

There are several studies indicating the protective effects of minocycline in the treatment of ICH. Minocycline has been reported to act as an iron chelator and an inhibitor of microglia activation [26, 27]. As a result, minocycline has been shown to reduce ICH-mediated brain iron overload and brain injury [26, 27]. Further, minocycline could also alleviate brain injury by inhibition of apoptosis and autophagy after ICH [28, 29]. To supplement those findings, the present study demonstrated that minocycline could suppress T cell infiltration and regulate CD4^+^ T cell activation [30, 31]. However, the exact mechanisms of minocycline function in the regulation of CD4^+^ T cells after ICH are still unclear. To the best of our knowledge, our current study delineates, for the first time, that minocycline is involved in the modulation of CD4^+^ T cell differentiation after ICH. We found that minocycline interacted with CD4^+^ T cells and accelerated CD4^+^ T cells to differentiate into Th2 and Treg cells, while suppressing the their differentiation into Th1 and Th17 cells and reducing neuroinflammation and white matter injury after ICH. Available literature has reported that Th17 cells can secrete IL17 and induce demyelination disorders [15, 32]. By inhibiting IL-17, inflammation could be attenuated. However, the function of IL-17 in ICH is unclear. Alternatively, regulatory T cells can inhibit macrophage/microglia activation and protect against secondary injury after ICH [11]. Our findings are consistent with this phenomenon. Therefore, minocycline could suppress neuroinflammatory response and attenuate white matter injury through modulation of CD4^+^ T cell activation and differentiation after ICH.

The current mechanisms of minocycline in regulating the differentiation and function of CD4^+^ T cells after ICH have not been delineated thoroughly. Notch has been implicated in almost all aspects of helper T cell differentiation, including the positive or negative modulation of T cell activation and differentiation to Th1, Th2, Th17, and Treg cells. Previous reports have demonstrated the role of Notch signaling pathway in regulating CD4^+^ T cell differentiation [13, 33]. The Notch signaling activation could disrupt the balance between CD4/CD8^+^ and Th17/Treg cells and incur inflammation [13, 14]. When the Notch1 signaling pathway was blocked with DAPT, the ratio of Th17/Treg was decreased, suggesting that the Notch1 signaling pathway plays an essential role in regulating the differentiation of Th17 cells and in maintaining the Th17/Treg balance [14]. Additionally, Notch1 signaling pathway could act as a bipotential switch, toggling between Th1 and Th2 cellular fates. Studies have suggested that Notch signaling could promote both Th1 and Th2 differentiation [34–36]. In a nutshell, our study has demonstrated that minocycline could activate Notch1 signaling pathway and modulate CD4^+^ T cell differentiation after ICH. After minocycline treatment, CD4^+^ T cells preferentially differentiated to Th2 and Treg cells after ICH, reducing neuroinflammation and white matter injury.

## 5. Conclusions

In conclusion, ICH leads to CD4^+^ T cell differentiation and neuroinflammation. Minocycline treatment could modulate CD4^+^ T cell differentiation and attenuate white matter injury via regulating Notch1 signaling pathway after ICH.

## Figures and Tables

**Table 1:** Primers used in the study.

Gene	Forward primer (5′ →3′)	Reverse primer (5′ →3′)
*β*-Actin	GCTTCTAGGCGGACTGTTACT	GCCTTCACCGTTCCAGTTTTT
IL21	AACAAGCAGAGATCCCGTGT	GCAGCAAACTCAGCAACCAA
IL22	GTGCGATCTCTGATGGCTGT	GACGATGTATGGCTGCTGGA
TNFa	AGCCGATGGGTTGTACCTTG	CTCCAAAGTAGACCTGCCCG
IFN -*γ*	GAGGTCAACAACCCACAGGT	GGGACAATCTCTTCCCCACC
TGF*β*	CTCCCGTGGCTTCTAGTGC	GCCTTAGTTTGGACAGGATCTG
IL-17	ACTACCTCAACCGTTCCACG	TTCCCTCCGCATTGACACAG

## Data Availability

All raw data used in this manuscript are available on reasonable request.
